# Evaluating the Perinatal Intensive Pathway: Reducing Hospital Admissions, Costs, and Enhancing Quality of Care

**DOI:** 10.1192/bjo.2026.11603

**Published:** 2026-06-30

**Authors:** Shambhavi Pranoy, Divya Sakhuja

**Affiliations:** Aneurin Bevan University Health Board, Newport, United Kingdom

## Abstract

**Aims:**

To evaluate the Perinatal Intensive Pathway’s effectiveness in reducing hospitaladmissions and crisis team referrals, and to explore patient, carer, and staff experiences, including potential cost savings to the local health board.

**Methods:**

Over a 12-month period, 12 patients were placed on the Perinatal Intensive Pathway, and required this support for at least two weeks. Quantitative data on avoided inpatient admissions and crisis team referrals were collected. Qualitative feedback was gathered via surveys from patients, relatives, and perinatal MDT members; thematic analysis summarised the experiences.

**Results:**

- *Quantitative findings:* Inpatient admission was avoided for all 12 patients (100%), and referral to the Home Treatment Team was avoided for 11 patients (92%). Within this NHS trust, an acute psychiatric inpatient bed-day costs**£607**per day, assuming an average admission of**14 days**, the PIP may have prevented approximately**£101,976**in inpatient costs in this small cohort.
- *Qualitative findings:* Patients and carers described the PIP as a “lifeline” providing reassurance, safety, and emotional and practical support. Improvements in confidence and wellbeing were frequently reported. Reported limitations were particularly around continuation of medication via General Practice, and communication of information to partners and carers. Staff reported benefits of earlier intervention, stronger therapeutic relationships, and reduced reliance on crisis services; they valued multidisciplinary input but noted gaps in out-of-hours support, variation in awareness of the pathway, and need for clearer written information. Feedback was overwhelmingly positive, with one patient calling the team “a God send.”

**Conclusion:**

The Perinatal Intensive Pathway successfully avoided hospital admissions and most crisis referrals, improved patient and carer experience, and delivered a notable cost saving by averting involvement of acute services. To maximise its impact, enhancements are needed in coordination with GPs, communication, and out-of-hours support. These findings support further implementation of PIP in perinatal mental health services.

